# A pictorial review of traumatic pericardial injuries

**DOI:** 10.1007/s13244-012-0177-9

**Published:** 2012-05-15

**Authors:** Ashok Adams, Nicos Fotiadis, Jann Yee Chin, Wayne Sapsford, Karim Brohi

**Affiliations:** The Royal London Hospital, Barts and the London NHS Trust, Whitechapel Road, Whitechapel, London, E1 1BB UK

**Keywords:** Pericardium, Heart injuries, Multidetector computed tomography, Ultrasonography

## Abstract

**Background:**

Thoracic injuries are the third most common injuries in trauma patients with cardiac injuries amongst the most lethal. Imaging is essential in diagnosis and triage of patients with pericardial injuries, and this review aims to highlight the spectrum of imaging findings of pericardial trauma. Focussed assessment with sonography for trauma (FAST) is the preferred initial examination, being rapid and accurate. Sensitivity of FAST for pericardial fluid detection is high with reported sensitivities of 97–100%. Plain chest radiography has low sensitivity for pericardial injuries but is useful in the evaluation of associated injuries. Computed tomography (CT) is the modality of choice for stable patients and can accurately diagnose traumatic pathology of the pericardium being especially useful in identification of cardiac herniation. The spectrum of CT findings includes pericardial fluid collections, focal pericardial defects and pneumopericardium.

**Methods:**

A selection of cases of pericardial trauma encountered at a level one trauma centre is presented. Operative findings were correlated with the FAST scan, plain radiography and computed tomography imaging.

**Conclusion:**

The imaging findings of pericardial trauma with various imaging modalities (ultrasound, plain radiography and computed tomography) are presented in order to aid interpretation during the acute trauma setting.

## Introduction

Injuries to the thorax are the third most common injuries in trauma patients [1] and have an overall fatality rate of approximately 10%. Cardiac injuries are among the most lethal in thoracic trauma patients, particularly in the setting of penetrating trauma [2]. The majority of patients who sustain penetrating cardiac injuries die at the scene with a reported survival rate as low as 6% [3]. The diagnosis of a pericardial injury relies on a high degree of clinical suspicion especially in patients with subtle imaging findings who may be haemodynamically stable on admission but subsequently deteriorate rapidly when a significant pericardial effusion develops. Dedicated imaging plays a cardinal role in the prompt management of these critically injured patients, and the aim of this pictorial review is to highlight the spectrum of imaging findings with a particular focus on cross-sectional imaging.

## Pericardial anatomy

The pericardial sac is formed by the pleuropericardial folds that grow from the lateral body wall in a coronal plane. These septae appear at the beginning of the fifth week of embryonic development as broad folds of mesenchyme that grow medially towards each other and eventually fuse to separate the pleural and pericardial cavities [4]. The pericardium contains the heart and juxtacardiac portions of the great vessels, and it is composed of an outer fibrous pericardium and an inner, double-layered sac, the serous pericardium [5]. The pericardial space lies between the visceral and parietal layers of the serous pericardium and contains approximately 15–50 ml of serous fluid produced by the mesothelial cells that line the serosa. This fluid facilitates freedom of movement of the heart within the pericardium.

Inferiorly, the fibrous pericardium is attached to the diaphragm’s central tendon. Anteriorly, the pericardium is separated from the thoracic wall by lungs and pleurae, but in a small area at the level of the 4th and 5th costal cartilages, it is in direct contact with the posterior aspect of the sternum. Laterally, the fibrous pericardium is related to the pleura and the medial pulmonary surfaces with the phrenic nerve descending between them. Posterior relations of the fibrous pericardium include the principal bronchi, oesophagus, descending thoracic aorta and the posterior portions of the mediastinal surfaces of both lungs. Superiorly, the fibrous pericardium blends externally with the great vessels, and it is continuous with the pretracheal fascia. Through its various attachments, the pericardium is securely anchored and maintains its thoracic position. A number of pericardial sinuses and recesses between the pericardial reflections serve as a potential space to accommodate a limited amount of fluid.

## Pericardial injuries

Cardiac injuries are more common in penetrating trauma compared to blunt trauma and are fatal in a significant percentage of patients with many found dead at the scene [2]. The anatomic position of the heart can account to some degree for the relative frequency of different injuries and, depending on the phase of respiration, penetrating abdominal injuries can also result in cardiac injury. The right and left ventricles are injured approximately 40% of the time, the right atrium approximately 24% of the time and the left atrium approximately 3% of the time [6].

Pericardial tears range in size from a few millimetres to several centimetres with extension along the entire length of the pericardium. Small pericardial lacerations often tamponade and the fibrous pericardium can seal with blood and clot accumulating within the pericardial space resulting in a haemopericardium. This may have a protective effect in preventing exsanguination into the thoracic cavity and indeed pericardiocentesis may remove the “tamponade” effect of collected blood on traumatised cardiac chambers resulting in circulatory collapse [6]. If the pericardial laceration is an isolated lesion, it is usually of no consequence [7], but when large enough, the heart may be dislocated and may result in torsion along an axis made by the inferior vena cava and the great vessels. Pericardial rupture is sometimes reported as an incidental finding at emergency thoracotomy/laporotomy but most are discovered post-mortem. The most common site of pericardial injury in blunt trauma is the left pleuropericardium parallel to the phrenic nerve [7]. Other common sites of pericardial injury, in descending frequency, include the diaphragmatic surface of the pericardium, right pleuropericardial surface and the superior mediastinal surface [8].

## Imaging of pericardial injuries

A number of imaging modalities are available for imaging the heart and pericardium in the acute trauma setting including ultrasound [FAST (focussed assessment with sonography in trauma) or echocardiography], conventional radiography and computed tomography (CT). The FAST scan includes a transverse subxiphoid view of the heart and right and left longitudinal views of each hemithorax. The sensitivity of FAST for pericardial fluid detection is reported to be as high as 97–100% particularly in dedicated prospective studies specifically evaluating the role of ultrasonography in pericardial injuries [9–11]. Operator experience is a potential limitation of FAST scanning, and other limitations of ultrasound in the assessment of heart and pericardial injuries include the presence of air within the pericardial sac and extensive surgical emphysema. In cases of severe thoracic trauma, extensive surgical emphysema tracking along the superficial soft tissues of the anterior chest wall limits the ability of FAST to assess for pericardial effusion as there is significant attenuation of the sound waves. In our centre, FAST ultrasound is performed immediately after the primary advanced trauma life support (ATLS) survey. In a series of 36 cases of surgically confirmed cases of pericardial trauma, the sensitivity of FAST for pericardial fluid detection was very high at 88% (unpublished data).

**Fig. 1a–c:**
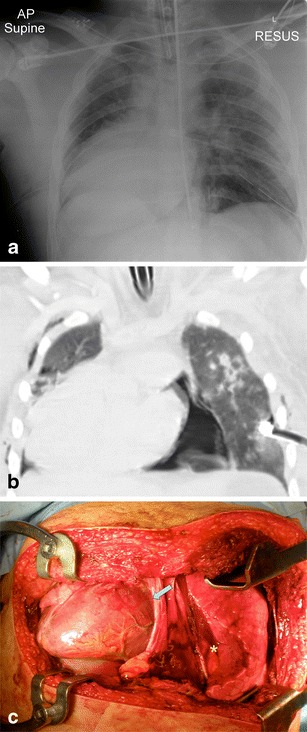
A 21-year-old male involved in a moderate speed motorbike accident with prominent transmission of force through the head and chest. Initial CXR (**a**) showing an endotracheal tube, oesophageal monitoring probe and bilateral tube thoacostomies. In addition a dextrocardia of uncertain significance was noted. The motorcyclist had sustained a right-sided pericardial tear with cardiac herniation into the right hemithorax. The selected coronal image on lung window setting (**b**) demonstrates the pneumopericardium with altered cardiac axis. On the intra-operative photo (**c**), note the expected position of the heart (*yellow asterisk*) and the site of the right-sided pericardial tear (*grey arrow*) which resulted in cardiac herniation. (**a** Reprinted with permission from [18])

**Fig. 2a, b:**
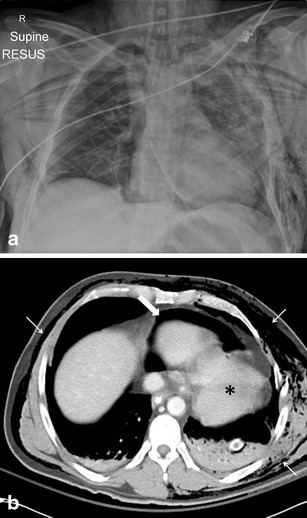
A 45-year-old male involved in a road traffic accident. **a** The chest radiograph identified the presence of pneumopericardium most evident adjacent to the right heart border with cardiac displacement. There are multiple injuries but note the gross surgical emphysema that limited the FAST assessment.**b** On the selected axial CT image on soft tissue window settings, there is extensive surgical emphysema (*thin white arrows*), pneumopericardium (*thick white arrow*) and note the displacement of the heart posteriorly into the left hemithorax (*black asterisk*). This patient had sustained a left-sided pericardial tear with cardiac herniation that was surgically repaired. (**a** Reprinted with permission from [18])

The chest radiograph has an established role in the initial trauma series, however it is limited in its ability to identify pericardial and cardiac injuries, and the main role of the chest radiograph is to identify other associated injuries (e.g. pneumothoraces, haemothoraces and pulmonary contusions). Plain radiograph findings that may portend a potential pericardial injury include an enlarged and/or globular cardiac outline (secondary to a pericardial effusion), pneumopericardium and alteration of the cardiac axis (Fig. 1a–c). With regards to a traumatic pericardial effusion, the presence of a normal cardiac silhouette occurs in up to 80% of trauma patients with acute tamponade as the pericardium has not had time to stretch [12, 13]. The accumulation of approximately 250 ml of fluid is required to enlarge the cardiac silhouette on a chest radiograph, but the accumulation of only 100–200 ml of fluid/blood within the pericardial space can result in physiological compromise and cardiac tamponade. The finding of pneumopericardium should alert the radiologist to consider loss of pericardial integrity. In severe cases, a tension pneumopericardium can develop with a resultant ‘small heart’ sign. Furthermore, the combination of pneumopericardium with displacement of the heart should alert the reporting radiologist to the possibility of cardiac herniation secondary to a pericardial tear (Fig. 2a–b). In the absence of pneumopericardium, displacement of the heart to the right or left without an obvious cause (e.g. compressive pneumothorax, haemothorax, air trapping in the lung or atelectasis) should also act as a warning sign for potential cardiac herniation [14–16]. It is also important to bear in mind that there are reports of delayed presentation of cardiac herniation as herniation has also been described following the institution or discontinuation of positive pressure ventilation [17]. Other findings that may suggest cardiac herniation include an unusual cardiac silhouette contour and a change in cardiac position after intercostal drain insertion [18].

Computed tomography (CT) imaging plays a crucial role in the assessment of stable patients with pericardial and cardiac injuries. Pneumopericardium can be difficult to differentiate from pneumomediastinum and medial pneumothorax on plain radiography alone. Axial or coronal CT images can aid differentiation if the plain radiography findings are difficult to interpret. If the pericardial tear is large enough, cardiac herniation can manifest on CT images through alteration of the normal cardiac axis (Fig. 1c), displacement of the heart (either within the left hemithorax or into the contralateral hemithorax), presence of air within an empty pericardium (“empty pericardial sac sign”) (Fig. 2b), and alteration of the normal cardiac contour (“collar sign”). The collar sign refers to a waist around the compressed portion of the heart herniating through a pericardial defect. Following injury to the diaphragmatic surface of the pericardium, there may also be herniation of bowel with bowel gas noted within the pericardial sac. Conversely, this may also result in cardiac herniation into the abdominal cavity [19]. From the various case series, these abdominal-pericardial tears are difficult to diagnose, and they have varied presentations. Furthermore, there are reports of delayed presentation of pericardial phrenic hernias ranging from weeks to years [20]. Other findings that may suggest cardiac herniation include an unusual cardiac silhouette contour. It is important to recognise that some of these features are seen in other conditions including ventricular aneurysms and ventricular chamber dilatation. Furthermore, partial or complete congenital absence of the pericardium can also result in cardiac axis deviation and pericardial discontinuity.

**Fig. 3:**
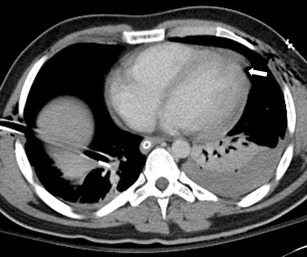
A 26-year-old male who sustained a stab wound to the left side of the chest. Note the focal pericardial defect at the cardiac apex (*white arrow*) overlying the left ventricle at the distal end of the stab wound track, which was confirmed intra-operatively

**Fig. 4:**
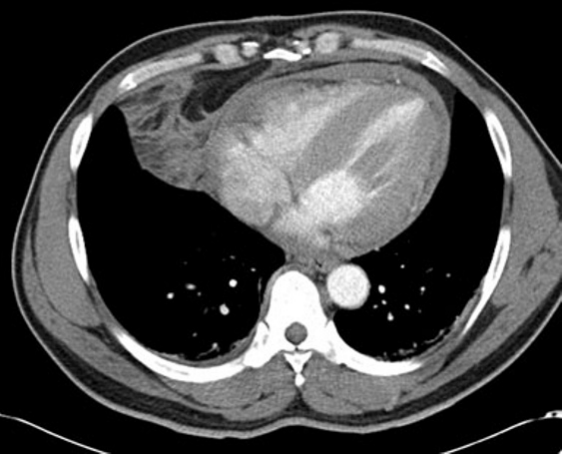
A 49-year-old male who sustained a stab wound to the epigastrium. The CT identified the presence of a pericardial fluid collection (approximate Hounsfield attenuation value of 40) and fat stranding within the epicardial fat. Intra-operatively this patient was confirmed to have a pericardial laceration and haemopericardium

Focal pericardial discontinuity can also be identified on CT images, and this can be direct indication of pericardial injury following blunt or penetrating trauma (Fig. 3). This can be subtle and easily overlooked, but these are important injuries to identify as patients can rapidly become haemodynamically unstable. Pericardial effusions are readily identified on CT and are another indicator of potential pericardial or cardiac injury (Fig. 4). Careful assessment is required particularly within dependent portions of the pericardial sac. The combination of haemorrhagic pericardial fluid, distended central veins (inferior vena cava, superior vena cava, hepatic, renal) with or without evidence of contrast reflux and periportal oedema may be suggestive of cardiac tamponade [21]. Other findings suggestive of cardiac tamponade secondary to a pericardial effusion include a compressive deformity of cardiac chambers, especially the more compliant right-sided chambers. There is resultant flattening of the anterior surface with a reduction in the AP diameter which has been described as the “flattened heart sign” [22].

Magnetic resonance imaging (MRI) has a potential role in the assessment of pericardial injuries in patients who survive the initial acute admission and are haemodynamically stable. The normal parietal pericardium is best visualised during systole on T1-weighted imaging, where it appears thin (<2 mm) and returns low signal intensity. However, the normal parietal pericardium is often incompletely visualised. Cardiac MRI has been utilised to confirm the diagnosis of pericardial herniation and cardiac rupture following blunt trauma [23]. In the case presented by Sohn et al. [24], cine MR images in a coronal plane (using a breathhold balanced fast field echo sequence) identified exaggerated up and down motion of the cardiac apex which was distinct from the left hemi-diaphragm. In addition, there was clockwise rotation of the cardiac axis, and the finding of a large pericardial tear with cardiac herniation was confirmed intra-operatively. Like CT, cardiac MRI can be used to assess the pericardium, but it has the advantage of being able to assess cardiac motion and to assess the functional significance of pericardial injuries and effusions.

## Summary

Imaging plays a cardinal role in the diagnosis and triage of patients with pericardial injuries. FAST ultrasound with a pericardial window is the preferred initial examination, being rapid and highly accurate. Plain radiography has a very low sensitivity for cardiac and pericardial injuries, but it is useful in the evaluation of associated injuries (e.g. pneumothorax, haemothorax). CT is the modality of choice for stable patients and can accurately diagnose a range of the traumatic pathologies of the heart and pericardium being especially useful in the identification of cardiac herniation.
